# RENEWAL: REpurposing study to find NEW compounds with Activity for Lewy body dementia—an international Delphi consensus

**DOI:** 10.1186/s13195-022-01103-7

**Published:** 2022-11-11

**Authors:** John T. O’Brien, Leonidas Chouliaras, Janet Sultana, John-Paul Taylor, Clive Ballard, on behalf of the RENEWAL Study Group, Dag Aarsland, Frederic Blanc, Bradley Boeve, David J. Brooks, K. Ray Chaudhuri, Jeffrey Cummings, Howard H. Feldman, Leon Flicker, James E. Galvin, Donald G. Grosset, Manabu Ikeda, Susan Kohlhaas, Brian Lawlor, Afina W. Lemstra, Iracema Leroi, Elisabet Londos, James B. Leverenz, Simon Lewis, Ian McKeith, Roger Mills, Richard Oakley, Jill Richardson, Marwan Sabbagh, John Skidmore, Per Svennigsson, Pietro Tiraboschi, Daniel Weintraub, Zuzana Walker, Rosie Watson, Rimona S. Weil, Caroline H. Williams-Gray, Alison Yarnall

**Affiliations:** 1 Department of Psychiatry, University of Cambridge School of Clinical Medicine, Cambridge, UK; 2 Cambridgeshire and Peterborough NHS Foundation Trust, Cambridge, UK; 3 College of Medicine and Health, University of Exeter, Exeter, UK; 4 Translational and Clinical Research Institute, Campus for Ageing and Vitality, Newcastle University, Newcastle, UK

## Abstract

Drug repositioning and repurposing has proved useful in identifying new treatments for many diseases, which can then rapidly be brought into clinical practice. Currently, there are few effective pharmacological treatments for Lewy body dementia (which includes both dementia with Lewy bodies and Parkinson’s disease dementia) apart from cholinesterase inhibitors. We reviewed several promising compounds that might potentially be disease-modifying agents for Lewy body dementia and then undertook an International Delphi consensus study to prioritise compounds. We identified ambroxol as the top ranked agent for repurposing and identified a further six agents from the classes of tyrosine kinase inhibitors, GLP-1 receptor agonists, and angiotensin receptor blockers that were rated by the majority of our expert panel as justifying a clinical trial. It would now be timely to take forward all these compounds to Phase II or III clinical trials in Lewy body dementia.

## Introduction

Dementia clearly represents a global and growing health challenge, estimated to affect over 100 million people worldwide by 2050. Lewy body dementia (LBD), the second commonest cause of degenerative dementia after Alzheimer’s disease (AD), accounts for around 10% of all clinically diagnosed cases [1, 2] and Lewy body pathology is present in up to 25% of dementia cases at autopsy. LBD includes two closely related conditions, dementia with Lewy bodies (DLB) when dementia develops before or within a year of onset of motor symptoms, and Parkinson’s disease dementia (PDD) when dementia occurs during the course of established Parkinson’s disease (PD) [3]. Both DLB and PDD are associated with very poor outcomes in terms of diminished quality of life [4], more rapid functional decline, and increased mortality compared to other dementias [5]. Limited symptomatic treatments exist, primarily cholinesterase inhibitors and memantine, but there are no disease-modifying treatments for LBD. Better treatments to improve these poor clinical outcomes are urgently needed.

The pathophysiology of LBD is complex. Alpha-synuclein deposition occurs intra-neuronally in the form of Lewy bodies and Lewy neurites, as in PD, and there are variable amounts of Alzheimer's type pathology, particularly non-neuritic amyloid plaques with a variable extent of tau tangle pathology. Other changes, such as neuroinflammation, are increasingly recognised to occur early in the disease [6, 7] and may impact the outcome, as has been shown for other dementias [8]. As such, potential strategies targeting disease modification may be directed at influencing α-synuclein deposition (either decreasing deposition, decreasing phosphorylation, or accelerating clearance), Alzheimer’s type amyloid and tau changes, or affecting the neuroinflammatory cascade.

DLB and PDD potentially represent a spectrum of disease, rather than discreet conditions, and treatment approaches for these integrated disorders for pharmacological and non-pharmacological management share much in common [9]. With the identification of several new potential treatment targets for LBD, there has been renewed interest from the biopharmaceutical industry in LBD clinical trials with several ongoing and some promising early results reported. For example, a Phase 2 study of neflamapimod, a mitogen-activated protein kinase (MAPK) inhibitor, which may regulate the endosomal protein Rab5 and modulate neuroinflammation by shifting microglial activation from a proinflammatory to a phagocytic state, has been shown to improve cognition in early reports [10, 11]. Despite this increased interest, there are only 14 ongoing Phase 2 or Phase 3 clinical trials of pharmaceutical interventions registered for DLB and PDD on the clinicaltrials.gov trial registry (compared to 158 studies for AD and >1800 for cancer), emphasising the urgent need to enhance the emerging treatment pipeline (https://clinicaltrials.gov/ct2/home, accessed 01/09/2022, search terms: Recruiting, Active, not recruiting, Enrolling by invitation Studies | Parkinson’s disease dementia OR Lewy body dementia OR Lewy OR Parkinson’s disease with dementia OR Parkinson-Dementia syndrome OR Lewy Body Parkinson dementia | Phase 2, 3).

An alternative to developing pharmacological agents de novo, at substantial cost and long lead-in time before clinical use, is to consider repositioning or repurposing of existing clinically available agents for new indications. This has been advocated for several conditions including cancer and other types of dementia [12, 13]. Many drugs, though developed for one target mechanism, have multiple pharmacological actions that may offer benefit in other conditions. Drugs that have already been approved by regulatory authorities or whose development was discontinued prior to approval have established dosing, tolerability, safety and side effect and well as manufacturing challenges, offering a significant reduction in development time for clinical trials. Many are off or nearing end of patent, thus offering the prospect of a widely available low-cost agent [12]. Drug repurposing has been defined as the application of established drug compounds to new therapeutic indications and offers a route to drug development that is accessible to academic institutions, government and research council programmes, charities and not-for-profit organisations thus complementing the work of pharmaceutical and biotechnology companies. Drug repositioning occurs within the biopharma industry during drug development and refers to the development of an agent for an indication other than the indication it was originally intended for. This new indication is prioritised during the development process and before approval [14]. Our study focused on drug repurposing.

With many potential candidates for repurposing, a key question is how to choose a compound or compounds with sufficient evidence to move forwards to clinical trials guiding both the scientific community and funders. A prioritisation process is important to achieve this and to gain both consensus and scientific credibility; such a process has been used in Alzheimer’s disease as previously prioritised compounds have been taken forward to clinical trials [15].

The aim of this study was, therefore, to undertake a robust prioritisation exercise to identify potential agents that might be suitable for repurposing for LBD (either DLB or PDD or both) and to assemble an international expert panel to provide a view on (a) whether there was sufficient evidence for a compound(s) to be taken forward into clinical trials and (b) if so, the compound’s priority order for further study. The intention was to develop an international consensus on the pathway forwards for clinical studies of repositioned and repurposed agents for LBD.

## Methods

We followed a Delphi consensus process to evaluate drugs with potential for repurposing for LBD. The Delphi consensus process has the advantage of combining targeted review of the evidence available with rigorous expert interpretation, including blinded input to avoid group think and bias, with a consensus approach to reach agreement. Therefore, it allows a standardised review of the evidence with a rating of priorities by the panel of experts to shortlist compounds for trials [13]. This review prioritised compounds for LBD comprising of both DLB and PDD through two rounds of a formal Delphi consensus development. The panel consisted of 35 international members with expertise from academia (31), the pharmaceutical industry (2) and the charity sector (2), including the authors of this review; participants are listed as study group authors.

In the first phase of the process, each panel member was asked to nominate up to ten candidate compounds, either licensed for use in other diseases or under development, for which they considered evidence supporting their potential to be therapeutically useful in LBD. For each compound, further questions included whether they would be useful for DLB, PDD or both. A full scoping review of the literature was prepared for compounds that were identified as high priority and were nominated by at least two panel members. The key factors included in the reviews included pharmacology, toxicology, brain penetration, preclinical, clinical and epidemiological data indicating potential for therapeutic use in LBD. The expert reviews utilised various resources and databases available such as the Drug Bank (https://go.drugbank.com/), the Electronic Medicines Compendium, the Food and Drug Administration, the Health Products Regulatory Agency and the Italian Drug Agency electronic resources. Information on the ability of the various drugs to cross the blood-brain barrier (BBB) and putative mechanisms were searched for in DrugBank, PubChem (https://pubchem.ncbi.nlm.nih.gov) and also using a broader literature search in PubMed. Drug Safety information was taken from the Summary of product characteristics available for each compound. Information concerning clinical trials was obtained from www.clinicaltrials.gov.

In the second phase of the Delphi process, the expert reviews were circulated to the panel asking for views on whether there was sufficient evidence to justify a clinical trial in LBD, if so for DLB, PDD or both, and if not, what further evidence was needed before such a trial could be justified. The experts were also asked to rank the candidates in order of priority based on the strength of the evidence with lower ranking score meaning higher priority for repurposing. General and compound-specific comments and views were recorded. All survey phases were conducted using an online survey platform (https://www.onlinesurveys.ac.uk/) for systematic recording and analysis of the responses. Reviews were prepared for compounds that were recommended for repurposing by at least two members of the panel at the first round of the process, and compounds that were recommended by at least 50% of the panel at the second round are discussed here.

## Results

### Delphi process

In the first round of the consensus process, a total of 70 approved compounds were recommended by the expert panel for repurposing disease-modifying trials. Nine candidate compounds or classes of compounds were prioritised by at least two members of the panel and were taken forward for expert reviews. These were ambroxol, the tyrosine kinase inhibitors nilotinib and bosutinib, the glucagon-like peptide-1 (GLP-1) receptor agonists liraglutide and exenatide, metformin, the angiotensin receptor blockers (ARBs) candesartan and telmisartan, fasudil, etanercept, rasagiline and salbutamol.

In the second round of the consensus process, ambroxol was ranked at the top of the priority list with 68% of panel members reporting that there was sufficient evidence for it to be taken forwards for a clinical trial in both DLB and PDD and an additional 11% suggesting that there was enough evidence for PDD only (see Table 1). From the remaining compounds, nilotinib/bosutinib, liraglutide/exenatide, metformin, candesartan/telmisartan and fasudil were ranked very closely in the list of priorities with a majority (50–60%) of the experts in the panel suggesting that there was sufficient evidence to justify a clinical trial with these compounds in both DLB and PDD.

**Table 1:** Results of the panel prioritisation

Compound	Average ranking	Yes for DLB and PDD	Yes only for DLB	Yes only for PDD
Ambroxol	2.6	68%		11%
Nilotinib/Bosutinib	2.7	60%	3%	3%
Liraglutide/Exenatide	3.3	58%	8%	8%
Metformin	3.7	54%	3%	8%
Candesartan/Telmisartan	3.8	57%	5%	8%
Fasudil	4.0	61%		6%
Etanercept	5.4	38%	3%	3%
Rasagiline	5.6	27%		14%
Salbutamol	5.9	30%		

The table provides the list of compounds prioritised by the Delphi consensus panel with the highest preference at the top. The consensus panel members were asked to nominate a list of up to ten compounds, rank them in order of preference and specify whether they believe the would be suitable for dementia with Lewy bodies (DLB), Parkinson’s disease dementia (PDD) or both. Ranking ranged from 1 to 9. Please note that in the average ranking the lowest the score reflects the highest preference for the compound to be taken forwards

### Summary of evidence reviews

#### Ambroxol

##### Mechanism and preclinical work

Ambroxol is a mucolytic agent used to break phlegm in respiratory infections as well as for relief of throat pain and is marketed in several European countries [16]. There is a growing body of preclinical evidence from cell and animal models of PD (6-hydroxydopamine-injected rats, transgenic mouse models and transgenic fly models) showing that ambroxol has neuroprotective effects through upregulation of glucocerebrosidase (GCase), which leads to reduction of α-synuclein pathology, and improvement of mitochondrial function [17].

Ambroxol has been found to act as a chaperone to GCase, is linked with the upregulation of GCase through the transcription factor EB pathway and acts by blocking autophagy, activating the secretory pathway and stimulating lysosomal exocytosis [18–22]. Ambroxol may promote correct post-translational protein folding, attenuate the unfolded protein response and rescue apoptosis by modulating cytochrome-C, caspase-9 and caspase-3 expression [20, 23, 24]. Ambroxol improves behavioural and motor deficits in animal models of PD and these improvements appear to be mediated by the attenuation of the effects of α-synuclein pathology and the recovery of the dopaminergic system [25–29]. Considering ambroxol’s effects on GCase, it has been studied in Gaucher’s disease. This is an autosomal recessive inherited disorder caused by homozygous mutations in the GCase encoding gene (*GBA1*) affecting multiple organs, and in some cases causing parkinsonism [30], whereas heterozygous *GBA1* mutations are the commonest genetic risk factor for PD [30]. In a study that used cultured macrophages from 14 Gaucher’s disease patients and PD patients with mono-allelic *GBA1* mutations, treatment of cultured macrophages with ambroxol augmented GCase activity in both patient groups [31].

##### Clinical studies

An open-label clinical trial of 17 patients with PD taking an escalating dose of 1.26 g of ambroxol daily for 186 days found that ambroxol crossed the blood-brain barrier and was detected in the cerebrospinal fluid (CSF) [20]. In the CSF, ambroxol was associated with a mean decrease in the activity of GCase by 19%, mean increase of GCase enzyme protein levels by 35% and mean increase of α-synuclein levels by 13% in patients with and without *GBA1* gene mutations [20]. The reduction in activity of GCase and increase in α-synuclein in the CSF may appear paradoxical; however, it is consistent with a decrease in activity of GCase in extracellular fluid due to the binding of ambroxol to the active site of GCase protein, enabling transportation to lysosomes within tissues where ambroxol will increase intracellular GCase activity [21, 29]. The increase of α-synuclein in the CSF may be interpreted as an increase of extracellular export of the protein from the brain tissues [20]. Ambroxol was found to be well tolerated and produced no serious adverse events. Considering that it penetrates the CSF and engages the treatment targets, the majority of the expert panel opined that this study provided evidence that ambroxol warrants further investigation in placebo-controlled trials to examine whether it can be a disease-modifying treatment in synucleopathies.

An observational study using an investigator-initiated registry followed off-label treatment with ambroxol of 38 patients with Gaucher’s disease and 3 PD patients with *GBA1* mutations for a median duration of 19 months (median dose of 435 mg/day) [32]. The investigators found that ambroxol in this group is safe and well tolerated [32]. It also showed preliminary evidence of clinical benefits including stable or improved neurological status, increased physical activity and reduced fatigue; however, these were based on subjective reports and not on standardised assessments [32].

##### Ongoing trials

At present, there are three ongoing Phase 2 placebo-controlled trials in LBD aiming to test the potential of ambroxol as a disease-modifying treatment. A trial aiming to recruit 75 participants with PDD [33] was expected to be completed in December 2021 [34] while there are two further clinical trials aiming to recruit 15 patients with LBD [35] and 172 patients with DLB [36] respectively that are ongoing.

Through the Delphi process, it was noted that while ambroxol shows excellent potential for future studies, it is likely that additional studies on pharmacokinetics and a better understanding of its mechanisms of action are needed. It is important to carry out more work in α-synuclein-driven mouse models for a more thorough investigation of the target and mechanisms. Interestingly, ambroxol has shown promise in animal models of amyotrophic lateral sclerosis (ALS) and is considered as a potential treatment for ALS as well [17, 37].

#### Nilotinib/bosutinib

##### Mechanism and preclinical studies

Nilotinib and bosutinib are tyrosine kinase inhibitors. They are both available on the market in the USA and the UK and licensed for chronic myelogenous leukaemia. Their putative mechanism of action in LBD is related to evidence showing that tyrosine kinases are upregulated in the brains of people with AD and PD [38]. Nilotinib and bosutinib have shown promising results in animal models of synucleinopathies, amyloidosis and tau hyperphosphorylation [39–41]. They improve behavioural and motor deficits in such models by increasing the clearance of α-synuclein, amyloid and hyperphosphorylated tau and by stimulating autophagy [42–45]. Tyrosine kinase inhibitors have an anti-inflammatory effect through modulation of various markers of neuroinflammation in animal models [40, 41]. Nilotinib was associated with rescued synaptic dysfunction [46]. Both nilotinib and bosutinib were found to prevent cell death due to trans-activating response of DNA/RNA-binding protein (TDP)-43 pathology, but only nilotinib reversed mitochondrial impairment [47]. There are, however, conflicting results as in a study of a mouse model of multiple system atrophy, in which nilotinib failed to reduce α-synuclein aggregate burden [48]. Although not directly measured, both nilotinib and bosutinib are predicted to cross the BBB [49].

##### Clinical studies

In clinical studies, the use of nilotinib in PD has so far yielded inconclusive results. A trial in 75 patients with PD randomised to receive placebo, nilotinib 150mg or 300mg for 12 months showed that nilotinib is safe, well tolerated and can increase dopamine metabolites in the CSF as well as reduce CSF α-synuclein oligomers and hyperphosphorylated tau [50]. An open-label extension of this study that included 63 patients for an additional 12 months randomised to receive 150mg or 300mg nilotinib showed that nilotinib continued to be safe and tolerated and demonstrated that nilotinib 300mg was associated with stable scores in the Movement Disorder Society Unified Parkinson’s Disease Rating Scale (MDS-UPDRS) from baseline to 27 months in parts I (nonmotor including cognitive) and II (activities of daily living), while nilotinib 150mg was associated with improvement in the sum of UPDRS Part I (nonmotor including cognitive) and II (activities of daily living) with no difference in the UPDRS Part III (motor examination) [51]. Quality of life measures also worsened in the nilotinib 150mg compared to 300mg group between 15 and 27 months [51]. However, another double-blind placebo-controlled study that enrolled 76 patients with PD who received nilotinib 150mg or 300mg or placebo for 6 months showed that, while nilotinib was safe and well tolerated, patients in the nilotinib arms showed worse motor scores (measured with the MDS-UPDRS) [52]. This study failed to identify any changes in the dopamine metabolites in the CSF and suggested that, at least for PD, there was no evidence to support further testing of nilotinib [52]. In parallel, in a double-blind study of nilotinib 150mg or 300mg or placebo in AD, nilotinib was overall well tolerated, although more adverse events, particularly mood swings, were observed with the 300mg dose [53]. This study showed that nilotinib was associated with reduced amyloid burden in the frontal lobes measured with amyloid positron emission tomography (Florbetaben PET), attenuated hippocampal atrophy on MRI and reduced CSF amyloid beta (Aβ) 40 and 42 and CSF phosphorylated tau 181; however, it was underpowered to detect any cognitive or clinical benefits [53]. With regards to clinical trials in LBD, a trial has been conducted in 12 patients with LBD randomised to nilotinib 150mg or 300mg for 24 weeks. This showed that nilotinib was safe and well tolerated and treatment increased levels of the dopamine metabolite homovanilic acid in the CSF [54]. A follow-up study involving analysis of CSF in these patients with PDD or DLB previously treated with nilotinib [50, 54] showed that nilotinib altered CSF microRNAs that regulate autophagy genes [55]. A further study from the same group randomised 26 participants with DLB to receive bosutinib 100mg orally or placebo for 12 weeks [56]. It showed that bosutinib was safe and well tolerated, penetrated the BBB to inhibit Abelson kinase and reduce CSF α-synuclein and dopamine catabolism. Secondary clinical outcome analyses showed that the bosutinib group had improved activities of daily living as measured with the Alzheimer’s Disease Cooperative Study- Activities of Daily Living (ADCS-ADL) but had no improvements in all other clinical, cognitive, neuropsychiatric and motor outcomes tested [56].

##### Ongoing trials

The results of two ongoing studies with nilotinib and bosutinib are awaited. These involve a Phase 1 trial of bosutinib in mild cognitive impairment (MCI) or dementia [57] and an ongoing placebo-controlled trial with 200mg nilotinib in LBD aiming to recruit 60 patients [58].

#### Liraglutide/exenatide

##### Mechanism and preclinical studies

Liraglutide and exenatide are synthetic GLP-1 analogues and GLP-1 receptor agonists. They are available in subcutaneous administration form and are approved for use in type 2 diabetes mellitus (T2DM) and weight loss in the European Union and the USA [59]. Liraglutide and exenatide have been studied extensively in preclinical models of AD and PD and there is evidence that exenatide can cross the BBB [60]. By acting as agonists of the GLP-1 receptor, they stimulate insulin release, inhibit glucagon release and delay gastric emptying. As such they are found to alter glucose metabolism in the brain, increase extracellular signal-regulated kinases (ERK) phosphorylation and decrease c-Jun N-terminal kinase (JNK) phosphorylation and thus prevent neurodegeneration [61–63]. Liraglutide decreases astrocyte and microglial activation, decreases chronic inflammation and lipid peroxidation, suppresses the apoptosis pathway and increases autophagy-related protein expression [64]. It reduces free oxygen species and increases the expression of glial-derived neurotrophic factor (GDNF) [65, 66]. It suppresses the protein kinase B/Glycogen synthase kinase 3β (Akt/GSK-3β) signalling pathway and acts on signal transducer and activator of transcription 3 (STAT3) to trigger cellular survival mechanisms [67, 68]. Liraglutide may decrease the formation of amyloid beta through effects on brain-specific human β-secretase 1 (BACE1) [69, 70]. In mouse models of AD, liraglutide rescued synapse loss and loss of synaptic plasticity in the hippocampus and was found to have a protective effect on brain vasculature [71]. Liraglutide was found to improve cognitive deficits in animal models of AD by reducing oxidative phosphorylation, oxidative stress, proinflammatory cytokines and neuroprotective effects [72–76]. Exenatide was found to preserve neurons in cellular and animal models of PD and attenuate the associated inflammatory response [77]. There is evidence that it can protect dopaminergic cells against metabolic and oxidative stress and prevent apoptosis, possibly by acting on caspase-3, mechanistic target of rapamycin (mTOR) and Akt signalling [78, 79]. Exenatide has also been found to reduce amyloid beta levels in several studies [80, 81]. Exenatide rescues choline acetyltransferase levels in a mouse model of AD, modulates Parkin and promotes the release of brain-derived neurotrophic factor (BDNF) [82]. Exenatide reverses age-related immune and energy metabolism transcriptomic changes as well as blood-brain barrier leakage in aged mice [83]. However, in a mouse model of prodromal PD, it led to an increase in pathological α-synuclein in brain regions connected to the olfactory bulb, accompanied by signs of microglial activation [84].

##### Clinical studies

There have been no clinical trials with liraglutide or exenatide in LBD. In PD, a double-blind, placebo-controlled trial in 62 patients with moderate PD assigned to have subcutaneous injections of exenatide 2mg or placebo once weekly for 48 weeks in addition to their usual medication, showed improvements in off-medication scores on Part III of the MDS-UPDRS by 1 point in the exenatide arm and worsening by 2.1 points in the placebo arm [60]. Secondary analyses in the same cohort showed that exenatide was associated with improvements in mood and depression measures while additional analyses in subgroups defined by age, motor phenotype, disease duration, severity, body mass index and insulin resistance showed that patients with older age of onset and with disease duration over 10 years responded less well to exenatide [85]. Subgroups with a tremor-dominant phenotype and lower MDS-UPDRS Part I scores at baseline experienced the best motor response to exenatide [86]. Another trial evaluated the progress of 45 patients with moderate PD treated with exenatide showed clinically important improvements in PD across motor and cognitive measures for exenatide compared to placebo. At 12 months, exenatide-treated patients had an average 2.7 point improvement on the MDS-UPDRS part III (motor examination) and a 2.8 point improvement in the Mattis dementia rating scale-2 in comparison with an average 2.2 and 3.5 point decline respectively in control patients [87]. Further follow-up of these trial participants 24 months after the first baseline visit (i.e. 12 months after stopping exenatide) showed that the exenatide group had an advantage of 5.6 points in the MDS-UPDRS Part III (motor examination) as well as an advantage of 5.3 points on the Mattis Dementia Rating Scale-2 [88]. A meta-analysis of these studies confirmed that exenatide is associated with improvement in cognitive, motor and nonmotor symptoms in PD [89].

Regarding studies testing the effects of GLP-1 analogues in cognitive disorders, a double-blinded placebo-controlled trial with liraglutide given for 12 weeks in late middle-aged individuals (age range 45–70) with subjective cognitive complaints, half of whom had a family history of AD, showed no cognitive benefits [90]. However, liraglutide was associated with improvement in intrinsic connectivity within the default mode network (DMN) measured with functional MRI (fMRI) [90]. A placebo-controlled study of liraglutide in 38 patients with AD showed no effects on Aβ levels or glucose metabolism in the brain measured with PET imaging [91]. One trial that had recruited 27 patients with early AD to receive exenatide or placebo was terminated early because the funder withdrew support. The reported results showed no group differences in clinical and cognitive measures, MRI measures or CSF biomarkers except for lower Aβ42 in plasma extracellular vesicles [92].

Meanwhile, a nationwide population-based case-control study found a statistically significantly decreased incidence of PD among diabetic individuals with a record of taking DPP-4 inhibitors (which increase GLP-1 levels) as well as a risk estimate below for GLP-1 agonists [93]. Another epidemiological study testing the association between prescription of various antidiabetic medications and PD found that the use of LG-1 mimetics is associated with a lower incidence of PD compared to the use of other oral antidiabetic drugs [94]. Similarly, a predictive algorithm using electronic health records to find associations between phenotypes and prescribed drugs found that prescription of liraglutide was associated with a decreased risk of a diagnosis of AD (adjusted OR 0.76) while a pharmacovigilance study aiming to compare the risk of AD among 66,085 patients with T2DM in the FDA spontaneous reporting database found that a prescription of exenatide or liraglutide was associated with a lower risk of developing AD compared to a prescription of metformin [95, 96].

##### Ongoing trials

There are no ongoing trials in LBD but there are trials investigating the potential benefits of liraglutide in AD and PD. A 12-month trial is testing the impact of liraglutide on cerebral glucose metabolic rate among 206 participants with AD [97] and another 6-month trial among 40 AD patients treated with liraglutide or placebo has been completed but the results are not yet reported [98]. An ongoing Phase 3 randomised placebo-controlled trial aims to investigate the efficacy of exenatide among 200 patients with PD over 96 weeks [99].

#### Candesartan/telmisartan

##### Mechanism of action and preclinical studies

Candesartan and telmisartan are ARBs widely used in cardiovascular disease as antihypertensives, as well as treatments for heart failure and left ventricular systolic dysfunction. They are predicted to cross the BBB [49]. With regard to putative mechanisms of action in LBD, they were found to inhibit the expression of Toll-like receptor 2 (TLR2) and candesartan has been shown to recue expression of both TLR2 and TLR4 in vitro and in a mouse model [100]. This is of particular relevance because both TLR2 and TLR4 are implicated in mediating the microglial response to α-synuclein in Lewy body disorders [101]. They have further been linked with reversing the activated proinflammatory phenotype of primary microglia reacting to oligomeric α-synuclein, and to reduce tumour necrosis factor alpha (TNF-α) levels [102–104]. Neuroprotective effects of these drugs may also include inhibition of the endoplasmic reticulum (ER) stress triggered by inositol-requiring enzyme/endonuclease 1α (IRE1α), downregulation of tumour necrosis factor receptor associated factor 2 (TRAF2) and activation of peroxisome proliferator-activated receptor (PPAR)- β/δ, as shown in a rotenone PD mouse model [105]. In a 1-methyl-4-phenyl-1,2,3,6-tetrahydropyridine (MPTP) mouse model of PD, telmisartan upregulated the expression of BDNF and GDNF and reduced markers of inflammation such as TNF-α and IL-1 [106, 107]. Regarding behavioural outcomes in animal models, candesartan and telmisartan have been found to improve motor deficits through increasing dopamine transporter markers and GDNF and through reducing levels of α-synuclein and attenuating ER stress-triggered neuronal apoptosis [107, 108].

Telmisartan and candesartan have variable effects on Αβ. Some studies have shown that they improve cognitive deficits in AD transgenic mice, prevent an increase of Αβ, phosphorylated tau and neprilysin, and decreased levels of TNF-α [109–114]. In contrast, other studies do not support use of candesartan or telmisartan in AD or PD due to limited benefits in animal models [115–117].

##### Clinical studies

No trial data are available in LBD. A study comparing candesartan, lisinopril and hydrochlorothiazide for 12 months in 53 individuals with cognitive problems but not dementia found that participants on candesartan had greater benefits in executive cognitive tests as well as improved preservation of cerebral haemodynamics [118, 119]. A follow-up study randomising 176 participants with MCI to receive candesartan or lisinopril for 12 months showed that candesartan was superior to lisinopril on the primary outcome of executive function measured with the Trail Making Test part B as well as several other tests [120]. A randomised placebo-controlled trial with candesartan that included 257 older adults with hypertension followed for a period of 44 months showed that candesartan was associated with less decline in attention and episodic memory but no differences in working memory or executive function, with effect sizes being in the small-to-moderate range [121]. Α randomised placebo-controlled trial of candesartan versus placebo in a total of 4964 elderly patients with mean follow-up of 3.7 years primarily aiming to measure cardiovascular events failed to show significant differences in Mini-Mental State Examination (MMSE) scores or proportion of patients who developed dementia [122]. In terms of epidemiological evidence, a prospective cohort analysis of a database of 819,491 male participants with cardiovascular disease showed that ARBs were associated with a reduction in the incidence and progression of clinically diagnosed AD and all-cause dementia compared to other antihypertensive and cardiovascular drugs [123].

##### Ongoing trials

There is one ongoing Phase 2 randomised placebo-controlled trial in 77 persons with MCI treated with candesartan for 12 months [124] and two ongoing trials concerning telmisartan in AD. The first is comparing telmisartan with perindopril in 150 MCI participants over 12 months [125], the other is testing whether telmisartan over 8 months has the potential to prevent AD in 66 African Americans who are at high risk of AD [126]. No studies are ongoing for LBD.

#### Metformin

##### Mechanism of action and preclinical studies

Metformin is a widely used anti-hyperglycaemic drug belonging to the biguanide class, mainly indicated for T2DM [127]. There is little evidence on putative mechanisms of action specifically for LBD and metformin is predicted to cross the BBB albeit with little probability [49]. In PD and AD animal models, metformin has been reported to lessen α-synuclein phosphorylation and aggregation, as well as astroglia and microglia activation, and shows neuroprotective effects [128–132]. Metformin prevented dopamine depletion, improved cell survival and promoted autophagy [133–136]. Furthermore, metformin inhibited oxidative stress, improved mitochondrial viability, reduced inflammation and improved synaptic function in several cell and animal models of PD and AD [137–141]. By reducing Αβ secretion and tau phosphorylation, it improved behavioural outcomes such as cognitive performance in several transgenic animal models of AD [129, 132, 142–144]. However, a study in a mouse model of tauopathy (ApoE−/−) reported an increase of Αβ formation and tau phosphorylation with metformin [145]. Metformin has shown beneficial effects in improving motor impairment in PD animal models [129, 146, 147]. There are however other animal studies where metformin was not associated with any benefits [145, 148].

##### Clinical studies

There is no specific evidence concerning use of metformin in LBD or PD. A randomised placebo-controlled pilot study testing metformin as a potential disease-modifying treatment for AD over 8 weeks with 20 subjects with MCI or mild AD dementia showed that metformin was associated with improved executive function providing some promising preliminary data for further research [149]. In another study, 80 overweight participants with amnestic MCI were randomised to receive metformin or placebo for 12 months [150]. Metformin was associated with modest improvements in the primary outcome of verbal learning and memory using the selective reminding test, but no changes were observed in the secondary outcomes such as the AD Assessment Scale-cognitive subscale (ADAS-cog), glucose uptake in the posterior cingulate-precuneus on fluorodeoxyglucose (FDG)-PET or in the levels of plasma Aβ42 [150]. A trial comparing the combination of metformin and donepezil to the combination of acarbose and donepezil in a total of 100 participants with abnormal glucose metabolism and non-dementia vascular cognitive impairment found that the group randomised to metformin-donepezil showed some cognitive improvements [151] on the ADAS-cog scale, the Trail Making Test and the World Health Organization University of California Los Angeles Auditory Verbal Learning Test. These cognitive benefits were associated with decreases in the levels of fasting insulin and insulin resistance, as well as lower common carotid artery intima–media thickness [151]. Meanwhile, several epidemiological studies have found associations between use of metformin and lower incidence of either all-cause dementia, AD or PD [152–157]. However, not all epidemiological studies have found associations between the use of metformin and a lower risk of dementia [158–160]. A study using the National Alzheimer’s Co-ordinating Center database investigated the effect of oral hypoglycaemic drugs on longitudinal memory decline among patients with T2DM with either normal cognition (*n*=1192) or with AD (*n*=807) and found that metformin was associated with better memory performance in non-demented participants (mean duration of follow-up 3.4 years) but it had no effects in AD (mean duration of follow-up 1.9 years) [161]. A systematic review and meta-analysis of observational studies testing the association between metformin and neurodegenerative diseases analysing a total of 19 studies with 285,966 participants found no association between metformin exposure and incidence of all subtypes of neurodegenerative diseases, and found that metformin monotherapy was associated with an increased incidence of PD compared to non-metformin or glitazone users (OR 1.66) [162].

##### Ongoing trials

There are no ongoing trials that are testing metformin in LBD. There are six registered trials testing metformin in MCI, early AD and patients with diabetes [163–168]. These trials test a variety of measures including cognitive performance as well as CSF and PET imaging markers of AD.

#### Fasudil

##### Mechanism of action and preclinical studies

Fasudil is a selective rhoA/rho protein kinase (ROCK) inhibitor. It is used in China and Japan for the treatment of vasospasm following subarachnoid haemorrhage [169]. Fasudil may act on LBD through several potential mechanisms. It crosses the BBB but due to limited bioavailability in the CNS a liposomal fasudil formulation for intrathecal injection was proposed to increase therapeutic efficacy and reduce side effects in an animal model [169–172]. It promotes the degradation of α-synuclein via autophagy through the c-Jun N-terminal protein kinase (JNK)1/Bcl-2/beclin 1 pathway [173]. Through reducing α-synuclein phosphorylation and total levels, preventing dopamine cell death and inflammation, fasudil improved motor deficits in various animal models of PD [174–176]. Fasudil was found to reduce microglial and astrocytic activation, increase GDNF and increase neuronal dendrite network organisation [177]. In AD animal models, fasudil rescued cognitive deficits and reduced acetylcholinesterase activity and oxidative stress [178–180]. Fasudil has been linked with promoting the release of neurotrophic factors and the dilation of cerebral vessels, inhibits the release of intracellular calcium, promotes axonal regeneration and reduces inflammation, Aβ deposition and tau phosphorylation [181–183].

##### Clinical studies and ongoing trials

There are no available clinical trials that have tested fasudil as a disease-modifying treatment in LBD, AD or PD. Furthermore, there are no registered ongoing trials testing fasudil in these conditions. There is an ongoing trial evaluating whether fasudil improves clinical outcomes in patients with ALS [184] and a Phase 2 trial of fasudil in patients with the 4-repeat tauopathies of progressive supranuclear palsy-Richardson syndrome or corticobasal syndrome [185].

##### Compounds not shortlisted

Etanercept, salbutamol and rasagiline were compounds that were recommended by more than one expert in the first phase of the Delphi consensus process but ranked low in the priorities in the second round. This was either due to the relative lack of preclinical evidence available or the absence of benefits in trials in AD or PD as none of these compounds have been tested in LBD. For example, rasagiline generally failed to show any cognitive benefits in PD patients with cognitive impairment [186–190]. Rasagiline has however shown some benefits in a double-blind parallel group placebo-controlled trial of 50 participants with mild to moderate AD randomised to receive 1 mg of rasagiline or placebo for 24 weeks. This trial showed favourable change in FDG-PET differences in rasagiline versus placebo in middle frontal, anterior cingulate and striatal regions along with benefits in measures of quality of life [191]. Meanwhile, rasagiline has shown some evidence in preclinical models in improving motor, cognitive and biochemical outcomes [192–194]. Similarly, limited evidence is available for the potential role of etanercept. Etanercept, a TNF inhibitor, has shown some promise in animal and cell culture models reducing cytokine release, preventing neurotoxic effects of TNF-α on dopaminergic cells and reducing caspase 3 activity [195–198], but had either limited or no benefits in trials in AD [199–201]. Finally salbutamol, a β2 adrenergic receptor agonist, has shown some benefits in preclinical studies improving cognition, preventing amyloid related changes, modestly decreasing a-synuclein levels and improving the viability of dopaminergic neurons in animal and cell culture models [202–204]. However, evidence from clinical studies is lacking, apart from one study showing that better control of asthma through treatment with salbutamol for 12 months improves cognition [205]. Studies in small cohorts in PD show modest global improvements while limited data are available from epidemiological studies with likely presence of confounding factors [206–212].

## Conclusions

We provide a comprehensive review of recently published and ongoing trials of agents potentially suitable for repurposing for LBD. Our initial prioritisation exercise identified nine candidate compounds or classes of compounds. In Table 2, we summarise the agents prioritised through our Delphi process, their proposed mechanisms of action, available evidence and future work required. As part of the methodology we followed for the Delphi consensus recommendations, we did not exclude compounds that are already in trials; therefore, ambroxol and nilotinib were highly prioritised in the process. While these trials are still ongoing, our Delphi consensus reviews show clear support for continuing research on the role of these compounds as disease-modifying treatments in DLB and PDD.

**Table 2:** Summary of agents prioritised for repurposing for LBD by the international Delphi panel

Proposed candidate	Suggested mechanisms of action	Summary of evidence	Future work required
Ambroxol	-Neuroprotective effects through upregulating GCase	-Reduction of α-synuclein pathology and improved mitochondrial function -Penetrates the CSF and engages the treatment target in humans	-Pharmacokinetics -Better understanding of mechanisms -Phase 2 work needed with CNS or CSF biomarkers to support target engagement
Nilotinib/bosutinib	-Increases the clearance of α-synuclein, amyloid, hyperphosphorylated tau -Stimulates autophagy -Anti-inflammatory effect -Rescues synaptic dysfunction	-Safe, well tolerated -Increase in dopamine metabolites in the CSF -Reduction of CSF α-synuclein oligomers and hyperphosphorylated tau -Worsening of motor scores	-Await results of ongoing trials -Safety work, especially in older adults and QTc prolongation
Liraglutide/exenatide	-Decreases astrocyte and microglial activation -Decreases chronic inflammation and lipid peroxidation -Suppresses the apoptosis pathway -Increases autophagy-related protein expression -Reduces free oxygen species	-Improvements in off-medication scores on part 3 of the MDS-UPDRS -Lower rate of PD compared to the use of other antidiabetic drugs	-Phase 2 work needed with CNS or CSF biomarkers to support target engagement in LBD
Candesartan/telmisartan	-Inhibits the expression of TLR2 and TLR4 -Reverses the activated proinflammatory phenotype of primary microglia -Reduces TNF-α levels	-Improvement of motor deficits in animal models -Reduction in levels of α-synuclein and attenuation of ER stress-triggered neuronal apoptosis -Improvement of cognitive performance in various cohorts	-More preclinical evidence and studies on whether they cross the BBB -Additional epidemiological evidence -Phase 2 trials in LBD
Metformin	-Prevents α-synuclein phosphorylation and aggregation -Prevents astroglia and microglia activation -Improves cell survival and promotes autophagy	-Reduction of amyloid beta secretion and tau phosphorylation -Improves cognitive performance in animal models. -Improves motor impairments in PD animal models -Improves verbal learning and memory in amnestic MCI.	-Phase 2 research needed with CNS or CSF biomarkers to support target engagement in LBD -Prodromal studies in enriched RBD may have a direct relevance for LBD
Fasudil	-Promotes the degradation of α-synuclein via autophagy through the JNK 1/Bcl-2/beclin 1 pathway -Dilates cerebral vessels -Inhibits the release of intracellular calcium	-Reduction of phosphorylated α-synuclein -Improves motor deficits in various animal models of PD -Rescues cognitive deficits, reduced acetylcholinesterase activity and oxidative stress in AD animal models	-Need clinical/pharmacokinetic studies to CNS penetration -Phase 1 clinical studies

All citations referring to the findings summarised in Table 2 are provided in the main body of the manuscript

*AD* Alzheimer’s disease, *BBB* blood-brain barrier, *Bcl2* B-cell lymphoma 2 protein, *CSF* cerebrospinal fluid, *CNS* central nervous system, *ER* endoplasmic reticulum, *GCase* glucocerebrosidase, *JNK* Jun N-terminal protein kinase 1, *LBD* Lewy body dementia, *MCI* mild cognitive impairment, *PD* Parkinson’s disease, *RBD* REM-sleep behavioural disorder, *TLR2* Toll-like receptor 2, *TNF-α* tumour necrosis factor alpha, *MDS-UPDRS* Movement Disorder Society Unified Parkinson’s Disease Rating Scale

Following detailed evidence-based review, over two thirds of the panel identified ambroxol as the top priority compound for both DLB and PDD, though even more (79%) thought there was evidence for PDD. Ambroxol, initially developed as a mucolytic agent, has activity as a molecular chaperone for the lysosomal enzyme GCase. Loss of function mutations in the *GBA1* gene that encodes GCase are one of the leading genetic risk factors for the synucleinopathies of PD and DLB [30]. This appears to be a highly promising compound for repurposing, and the largest planned trial is a Phase 2 Norwegian study of people with dementia or MCI with Lewy bodies that will enroll 172 participants [36]. Further trials are warranted.

Nilotinib and bosutinib are tyrosine kinase inhibitors and have shown promising preclinical evidence of effects both on α-synuclein and hyperphosphorylated tau as well as evidence of tolerability. However, during the course of this Delphi panel study, a double-blind placebo control Phase 2 study over 6 months in people with PD suggested that those on nilotinib had worse motor scores than placebo and, importantly, there was no evidence of central CNS penetration as dopamine metabolites in CSF did not change [50]. This suggests that, of the two compounds, bosutinib may be more worthy of further investigation than nilotinib.

There was support for the GLP-1 receptor agonists liraglutide and exenatide, and both are being assessed in AD trials, while exenatide is undergoing a Phase 3 trial in PD. Subcutaneous administration is required which is clearly less convenient than oral therapy, but trials of other agents administered subcutaneously or even by intravenous infusion have proved acceptable in AD. While not the focus of this repurposing study, oral GLP-1 receptor agonists are becoming available (e.g. semaglutide) which would be an easier dosing route for future studies [213], while alogliptin, an oral compound inhibiting DPP-4, the enzyme that inactivates GLP-1, thus boosting GLP-1 indirectly, is also trialled in PD [214].

Candesartan and telmisartan are ARBs, in wide clinical use as antihypertensives and for heart failure. They have a number of potential actions of relevance in LBD including actions on microglia and the endoplasmic reticulum. No trials are ongoing or planned in LBD, though there are ongoing trials in MCI and AD. There is good rationale for examining these compounds in LBD.

Metformin is a widely used anti-hyperglycaemic drug which has been shown to prevent α-synuclein phosphorylation and aggregation in animal models and prevent astroglial and microglial activation. There are ongoing studies in MCI and AD, but no identified studies in LBD. The panel concluded that studies of metformin in LBD are therefore warranted.

Fasudil is a selective rhoA/kinase (ROCK) inhibitor used for the treatment of subarachnoid haemorrhage in China and Japan. There are ongoing studies in progressive supranuclear palsy and ALS, but no studies in AD or LBD. Notably fasudil was prioritised in a recent Delphi consensus study of repurposing in AD [13].

In summary, through an international Delphi study, we have identified several promising compounds that have sufficient evidence to be taken forward into Phase 2 and Phase 3 studies for LBD. Given the current lack of any disease-modifying therapies and the huge burden of disease globally, both in terms of numbers affected and adverse impact on quality of life and mortality, there is a clear and urgent need to undertake clinical trials of these compounds in LBD.

## Data Availability

Not applicable.
